# Intratumoral Heterogeneity in Differentiated Thyroid Tumors: An Intriguing Reappraisal in the Era of Personalized Medicine

**DOI:** 10.3390/jpm11050333

**Published:** 2021-04-23

**Authors:** Antonio Ieni, Roberto Vita, Cristina Pizzimenti, Salvatore Benvenga, Giovanni Tuccari

**Affiliations:** 1 Department of Human Pathology in Adult and Developmental Age “Gaetano Barresi”, Section of Pathology, University of Messina, 98125 Messina, Italy; tuccari@unime.it; 2 Department of Clinical and Experimental Medicine, University of Messina, Viale Gazzi, 98125 Messina, Italy; roberto.vita@unime.it (R.V.); sbenvenga@unime.it (S.B.); 3 Department of Biomedical, Dental, Morphological and Functional Imaging Sciences, University of Messina, Viale Gazzi, 98125 Messina, Italy; cristina.pizzimenti@unime.it; 4 Master Program on Childhood, Adolescent and Women’s Endocrine Health, University of Messina, Viale Gazzi, 98125 Messina, Italy; 5 Interdepartmental Program of Molecular & Clinical Endocrinology and Women’s Endocrine Health, University Hospital, A.O.U. Policlinico G. Martino, Viale Gazzi, 98125 Messina, Italy

**Keywords:** intratumoral heterogeneity, thyroid tumor, BRAF, RET/PTC rearrangements, RAS mutation

## Abstract

Differentiated thyroid tumors (DTTs) are characterized by significant molecular variability in both spatial and temporal intra-tumoral heterogeneity (ITH), that could influence the therapeutic management. ITH phenomenon appears to have a relevant role in tumor growth, aggressive behavior and drug resistance. Accordingly, characteristics and consequences of ITH in DTTs should be better analyzed and understood in order to guide clinical practice, improving survival. Consequently, in the present review, we investigated morphological and molecular ITH of DTTs in benign, borderline neoplasms and in malignant entities, summarizing the most significant data. Molecular testing in DTTs documents a high risk for recurrence of cancer associated with BRAF^V600E^, RET/PTC 1/3, ALK and NTRK fusions, while the intermediate risk may be related to BRAF^K601E^, H/K/N RAS and PAX8/PPARγ. In addition, it may be suggested that tumor genotype is associated with peculiar phenotype.

## 1. Introduction

**Figure 1:**
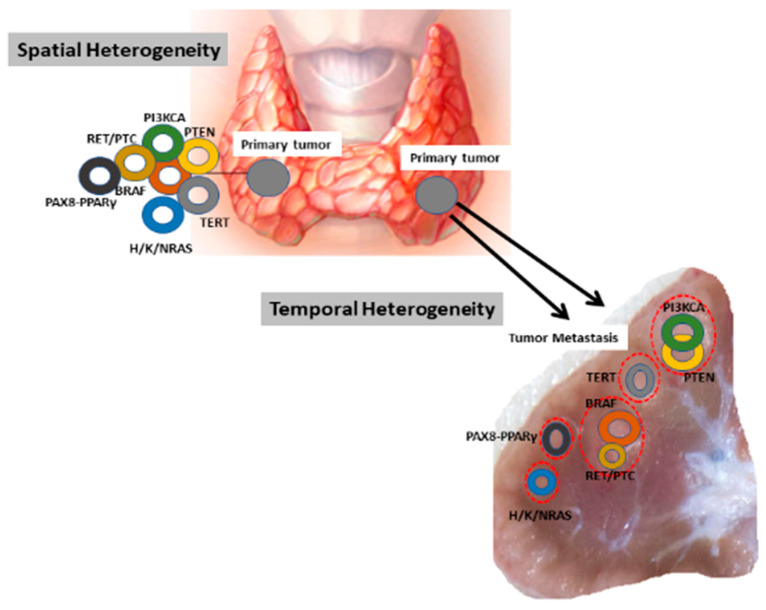
Spatio-temporal heterogeneity in primary and corresponding lymph node metastasis in thyroid tumors.

Intratumoral heterogeneity (ITH) represents a crucial determinant to explain the appearance of therapeutic resistance and treatment failure, resulting in poor prognosis and outcome. This intralesion mechanism is defined as diversity observed within a tumor since mosaics of different neoplastic clones are present in the same tumor at varying time [1]. ITH can exist either between geographical areas of the same tumor (spatial heterogeneity) or between different lesions that appear over time locally or distantly (temporal heterogeneity) (Figure 1) [1,2].

**Figure 2:**
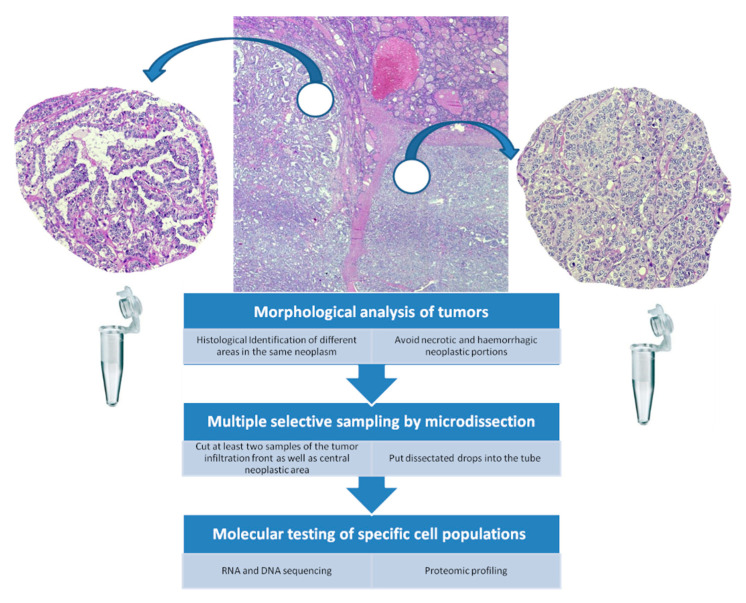
A schematic proposed workflow to identify ITC and to perform molecular tests in DTC.

Temporal ITH leads to discordance between the primary tumor and the metastatic lesion, and it can stem from either two mutations in different clones in the primary tumor, one clone disseminating to the metastatic site or from a new mutation occurring in the metastatic lesion [3]. ITH may determine the development of different cell subpopulations which in turns may influence the response of a tumor to changes within the microenvironment [3,4]. In addition, this phenomenon may create a neoplastic diffusion throughout the body, realizing metastatic deposits or acquiring resistance to therapeutic agents. Therefore, ITH analysis can provide relevant information to define innovative and patient-tailored therapeutic strategies, based on detection of specific molecular alterations [2,4,5,6]. So far, ITH has been addressed at both morphological and molecular levels with different methods [7,8,9,10]. Specifically, microdissection is essential to define morphological heterogeneity, which includes histotype, tissue composition, inflammatory reaction, center and borders of tumors [6,8]. Indeed, at a microscopic level, pathologists can recognize different histological patterns in each tumor, with specific morphological characteristics, such as necrosis, apoptosis, fibrosis, hemorrhagic areas, stromal reaction and neo-angiogenesis. An operative workflow to analyze ITH in tumors should be based on morphology, requiring an examination of extensive neoplastic areas to identify the different histological portions of the tumor, analyzing at least two or more representative different portions obtained by microdissection procedure (Figure 2). However, the intratumoral differentiation (well-, moderately-, poorly-) is frequently focal, leading to morphofunctional differences. Furtherly, the choice of tissue specimens may depend on the location of the tumor infiltration front, with elements able to invade capsule and stroma, in contrast to central neoplastic section (Figure 2). Then, the dissected portions have to be passed in a tube for the RNA/DNA sequencing as well as proteomic profiling (Figure 2).

In addition to the inter-tumor heterogeneity, namely diversity between individuals having the same tumor type, ITH may result in different histological and cytological patterns in the same tumor, negatively impacting on the patient’s prognosis [11,12,13]. At the molecular level, genetic and epigenetic heterogeneity can be present [14]. Particularly, immunohistochemistry, in situ hybridization methods and next generation sequencing by detecting mutations of the driver genes secondary to genetic instability may help reveal clonal and/or non-clonal heterogeneity, which are associated to phenotypic alterations driving neoplastic progression and resistance to targeted therapy [6,15,16,17,18,19].

One of the most evident examples of ITH is encountered in thyroid carcinomas and adenomas. Although the majority of differentiated thyroid carcinomas (DTCs) show an indolent behavior with an excellent prognosis, as documented by a 10-years survival rate of 90%, approximately 10% of them are aggressive, tend to recur and lead the patient to death [20,21,22,23,24]. In this regard, a broad ITH is evident, with histotypes spanning from thyroid papillary microcarcinoma through anaplastic carcinoma, the latter representing the late and fatal stage of carcinogenesis [25,26,27,28,29].

The present paper should be considered as a review in order to furnish the “state of art” regarding ITH in differentiated thyroid tumors (DTCs). The major endpoint is to comparatively analyze morphological and molecular ITH of differentiated thyroid tumors (DTTs), either follicular adenoma (FA) either DTCs in order to evaluate their behavior, identifying markers for therapeutic approaches and making individualized their management. Regarding the novelty of the present review, probably there are not additional original data, but a relevant number for information concerning molecular variability in DTCs in relation to the corresponding morphological aspects as well as a precise definition of the operative workflow to reveal ITH.

## 2. Phenotypic and Molecular Heterogeneity in FA and Follicular-Patterned Borderline Lesions

**Figure 3:**
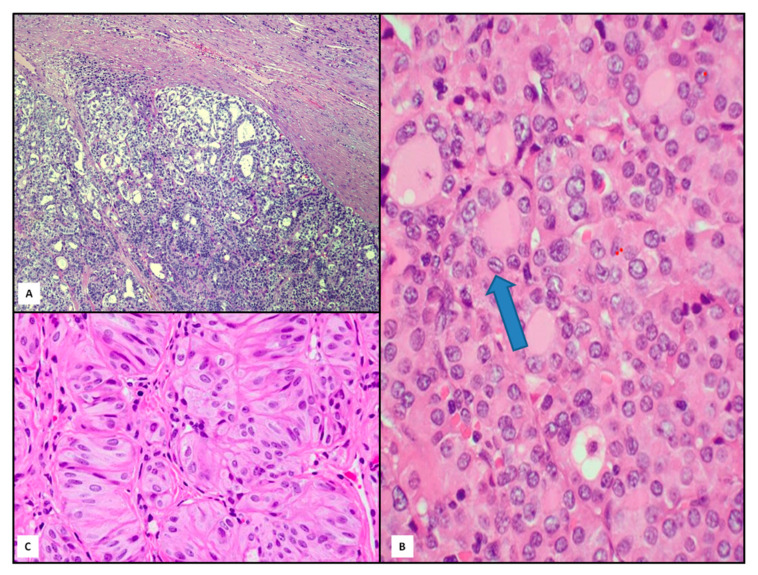
Histological findings of follicular-patterned borderline lesions: tumor with uncertain malignant potential (UMP) ((**A**), hematoxylin and eosin, 80×), noninvasive follicular thyroid neoplasms with papillary-like nuclear features (NIFTP) ((**B**), hematoxylin and eosin, 400×) and hyalinizing trabecular tumor (HTT) ((**C**), hematoxylin and eosin, 200×). The arrow underlines the peculiar irregular nuclear membranes.

To define morphological ITH in DTTs, we need to introduce the new classification of thyroid tumors, in which some changes were introduced [30,31,32,33,34]. In fact, together with follicular adenoma (FA), some borderline entities were added, such as tumors with uncertain malignant potential (UMP), noninvasive follicular thyroid neoplasms with papillary-like nuclear features (NIFTP) and hyalinizing trabecular tumor (HTT) [30,32] (Figure 3). FA were defined as benign, encapsulated and non-invasive neoplasm demonstrating evidence of thyroid follicular cell differentiation without nuclear features observed in papillary thyroid carcinoma (PTC) [30]. According to the 2017 WHO classification [35], the group of encapsulated follicular-patterned UMP tumors is divided into two entities: follicular tumors with uncertain malignant potential (FT-UMP) and well-differentiated tumors with uncertain malignant potential (WDT-UMP) (Figure 3A). FT-UMP is an encapsulated and/or well-circumscribed tumor with round nuclei that lack PTC-like features, whereas WDT-UMP presents a similar gross morphology but, well/partially developed nuclear changes of PTC and questionable capsular or vascular invasion [36,37].

NIFTP is a solitary encapsulated nodule displaying the following features: a complete, frequently thick, fibrous capsule delimitating the tumor from adjacent tissue, follicular growth pattern and nuclear features of PTC [38] (Figure 3B). Papillae and capsular or vascular invasion are constantly absent [38,39]. Furthermore, NIFTP may be distinguished from both FA and hyperplastic nodule by the presence of the typical nuclear changes of PTC [40,41,42,43] (Figure 3B).

Another new interesting follicular-derived borderline lesion is represented by HTT, a well circumscribed solid neoplasm without capsular/vascular invasion or invasion of thyroid tissue adjacent to the tumor. Histologically, HTT is composed of trabeculae or sometimes nests of polygonal eosinophilic large cells intermingled with thin stromal bundles. This lesion maybe associated with chronic thyroiditis, nodular goiter or PTC [44,45,46] (Figure 3C).

Molecular profiles concerning classical FA and follicular-patterned borderline tumors are quite different (Table 1).

For instance, paired box gene 8 (PAX8)-peroxisome proliferator-activated receptor-γ (PPARγ) rearrangements are detected in about 5–20% of FA, but they are absent in non-pathological thyroid parenchyma surrounding FA or in the hyperplastic nodules [47,48,49]. Of the other somatic genetic alterations, Eukaryotic Translation Initiation Factor 1A X-Linked (EIF1AX) gene activating mutation is found in 5–10% of FA [50,51], while telomerase reverse transcriptase (TERT) promoter mutation are very rare in genuine FA and occasionally present in FA with atypical features [50,52]. Additionally, mutations concerning Enhancer of zeste 1 polycomb repressive complex 2 subunit (EZH1) gene are detected in 3% of the FA, frequently in association with the TSH-receptor (TSHR) and/or the guanine nucleotide binding protein, alpha stimulating (GNAS) mutations, and accounting for nearly 80% of cases in some series [33,47,53]. RAS mutations exhibit different rates in FA: mutations in HRAS are detected in 8%, in NRAS in 6% and in KRAS in 10%, respectively [51,54,55]. Interestingly, RAS mutations have a higher prevalence in FA of persons living in area of iodine deficiency [56]. In UMP tumors, HRAS mutation are present in 3–12% of cases at codon 61, similarly to KRAS mutations (6–9% of cases), but less frequently than NRAS mutations (16–35% of cases) [50,51,54,55]. However, H/N/K-RAS mutations are detected in 45% of NIFTP cases, while BRAF^V600E^ mutation and Rearranged during transfection (RET) fusions are absent [51,55]. Finally, HTT lacks BRAF or NRAS mutations, but it has considerable frequency of RET/PTC rearrangements (47%) similar to that encountered in PTC [55,57].

## 3. Phenotypic and Molecular Heterogeneity in FTC

**Figure 4:**
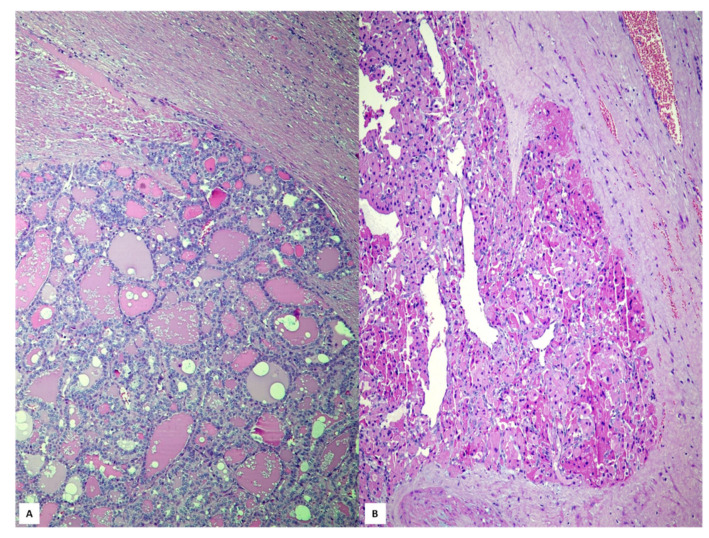
The follicular thyroid carcinoma with minimally invasive capsular infiltration ((**A**), hematoxylin and eosin, 80×); malignant Hürthle cell tumor characterized by an evident capsular invasion ((**B**), hematoxylin and eosin, 100×).

Follicular thyroid carcinomas (FTCs), which are well-known more aggressive cancer compared with PTCs, have a prevalent histologic presentation as microfollicular or trabecular patterns, and a less frequent architecture with follicular and colloid-rich morphology [58,59]. There are also morphological rarer subtypes that are predictive of a worse prognosis, including spindle cells, clear cells, signet-ring cells, rhabdoid and insular phenotypes [60,61]. Generally, regardless of histotype, FTCs present a thin or thick fibrous capsule that contains some small vessels; consequently, capsular invasion produces an incomplete delimitation of the tumor and becomes an indicator of vascular invasion. Based on the extent of capsular/vascular invasion, FTCs may be divided into two subgroups of prognostic significance, minimally invasive FTCs (Figure 4A) and widely invasive FTCs [60,61]. In 2015 the prognostic subgroups became four: minimally invasive with capsular invasion, minimally invasive with limited [<4 vessels) vascular invasion, minimally invasive with extensive [≥4 vessels) vascular invasion and widely invasive [59,62]. Recently, the last 2017 WHO classification suggested a 3-tiered risk groups: minimally invasive [capsular invasion only), encapsulated angio-invasive and widely invasive [59,60,61,62,63].

From a molecular perspective, the major driving mutations of FTCs are those in the RAS family of genes; for this reason, these tumors are also known as RAS-like tumors [30,51,64,65] (Table 2).

In detail, the three concurrent somatic H/N/K-RAS mutation were detected with different percentage, 8%, 19% and 6% respectively [51]. In addition, TERT promoter (TERTp) mutations have been revealed in 25% of FTCs, which are characterized by older age of patients, larger tumor size, advanced stage (III–IV), distant metastases and disease specific mortality [52]. Several other genes, which are generally involved in Phosphoinositide 3-kinases (PI3K)/Phosphatase and tensin homolog (PTEN)/AKT pathway have been found as mutated in FTCs [66,67]. PIK3CA copy gains are encountered in FTCs (10%) in comparison to BRAF mutations (1%) [67,68]. PTEN and PIK3CA mutations as well as PIK3CA copy gains rarely coexist in FTCs, while PI3K-PTEN-AKT pathway is common in poorly differentiated and anaplastic thyroid carcinomas, suggesting their important role in tumor progression [68,69]. Moreover, in FTCs EIF1AX mutation was identified in 6% of cases related with advanced disease [67,68]. ITH was also detected in FTCs with a range of histologic aspects, namely with follicular areas coexisting with poorly differentiated ones [67]. Interestingly, in poorly differentiated aggressive FTCs are characterized by frequent mutations in p53 (10–30%), RAS (10–40%), BRAF (5–30%) [69,70], but rare PAX8/PPARγ rearrangements (7–10%) have been reported [71,72]. Nevertheless, in FTCs PAX8/PPARγ rearrangements were revealed in female and younger patients with high cellularity and invasive aspects; this positively rearranged FTC documented a lower risk for distant metastasis [50].

Even if classified as follicular-patterned tumors, Hürthle cell tumors (HCTs) present peculiar microscopic characteristics consisting in large elements with abundant eosinophilic granular cytoplasm, centrally located nuclei and prominent nucleoli [73]. The new WHO classification distinguishes benign and malignant HCTs on the basis of capsular and vascular invasion (Figure 4B), similarly to FTCs [73]. Although believed to have a poorer prognosis compared to FTCs, it was demonstrated that Hürthle cell cancer has not higher rates of recurrence and does not concentrate less radioiodine [74,75,76]. Nevertheless, it has been reported that somatic genomic alterations in malignant HCTs are represented by Mucosal Vascular Address in Cell Adhesion Molecule 1 (MADCAM-1) (20%), EIF1AX (11%), DAXX, PT53 (7%) and Neurofibromatosis type 1 (NF1) (7%) mutations, while no BRAF mutations and a lower rate of NRAS (9%) mutation are encountered in comparison to FTC cases [77,78,79] (Table 2). In addition, TERTp and KRAS mutations have been identified in 17% and 11% respectively; NRAS occurred with a lower percentage (6%) [51].

## 4. Phenotypic and Molecular Heterogeneity in PTC and Its Variants

**Figure 5:**
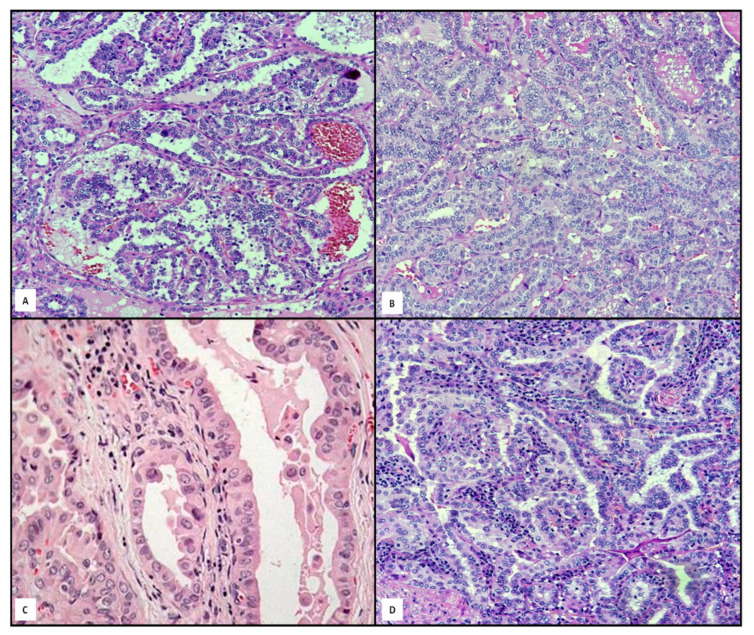
A gallery of some relevant variants of PTC: classical ((**A**), hematoxylin and eosin, 120×); follicular ((**B**), hematoxylin and eosin, 120×); Whartin-like ((**C**), hematoxylin and eosin, 120×); micropapillary ((**D**), hematoxylin and eosin, 160×).

As well known, PTCs are not only the most common DTCs, but also the most common malignant entity, in that they account for over 70% of all thyroid neoplasms [80]. The classical variant presents typical microscopic features (Figure 5A), such as overlapping and clearing nuclei, irregularities of nuclear membrane in papillary architecture with fibrovascolar cores, psammoma bodies and sometimes aggregates of lymphocytes. Sixteen PTC variants with different behavior have been reported so far [81,82,83,84,85,86]. After the classic variant, the most common variants are the follicular one (Figure 5B), hobnail/micropapillary (Figure 5C), Whartin-like (Figure 5D) and solid one [81,82,83]. In spite of this morphological variability, molecular ITH is not constantly present, even in multifocal PTCs [84,85,86].

Genetic ITH of PTCs was scarcely addressed so far, partly due to the relatively low number of oncogenes involved in the early stages [87,88,89] (Table 3). BRAF mutations have been reported in 55% of the classical phenotype with a further significant increase in more aggressive and poorly differentiated PTCs [90,91], and in up to one third of cases of the columnar-cell variant [92,93]. In addition, BRAF mutations are frequently combined with TP53, TERTp, PIK3CA, catenin β-1 (CTNNB1), epidermal growth factor receptor (EGFR), v-akt murine thymoma viral oncogene homolog 1 (AKT1) and Notch homolog-1 (NOTCH1) mutations [83,94,95,96]. In the hobnail variant, the mutations detected concern BRAF (25%), TP53 (55%) and NOTCH1 (5%) [83,94,95,96]. It is well known that mutations in BRAF and RET genes (see below) may occur both in the initial steps of carcinogenesis and in the advanced ones [97,98]. However, different foci of the same PTC may differ for their BRAF status, and such difference may also exist between a primary PTC and any of its lymph node and/or distant metastases in up to one third of cases [97,98,99]. For instance, BRAF^V600E^ mutation may occur either *de novo* in metastasized lymph nodes, or in metastasizing mutated cells could spread from non-analyzed PTC foci of the primary tumor [100,101,102]. ITH in BRAF^V600E^ have been also demonstrated, since only less than 50% of neoplastic elements manifested BRAF mutation [103,104]. Although, the prognostic role of BRAF^V600E^ mutation is still debatable, some studies showed an association with poor outcome, extra thyroid neoplastic extension and increased recurrence risk in PTCs [50,96,97,98]. By contrast, PTC with low risk clinicopathological features did not exhibit BRAF^V600E^ mutations [101,102,103].

Another common genetic alteration in PTCs is the RET/PTC rearrangement [105,106], which occurs in one third of cases of sporadic PTCs in adults, in half of cases of PTCs in children and young adults, mainly when lymph node metastasis and aggressive clinicopathological features were documented, similarly to NTRK rearranged PTC [107,108,109]. However, it has been shown that PTCs characterized by fusion oncogene (RET or NTRK) exhibited overlapping clinical behavior [109]. RET/PTC rearrangement has been also frequently observed in subjects exposed to radiation, either accidentally or therapeutically [110]. Moreover, this genetic alteration early occurs in thyroid carcinogenesis, being essentially restricted to PTCs and Hürthle cell tumors [111,112]. Finally, a low rate (1–5%) of PTCs documented ALK rearrangement in predominant follicular solid infiltrative pattern or in diffuse sclerosing variant, showing sometimes extrathyroidal extension as well as lymph node metastases [113].

As said at the beginning of this review, different methods of detection (immunohistochemistry, RT-PCR, RNA analysis after Laser Capture Microdissection) are able to detect RET/PTC rearrangements, the distribution of which may be influenced by intrinsic genetic [111]. Interestingly, Schopper et al. tested a panel of 8 cancer-related genes (BRAF, KRAS, HRAS, NRAS, EGFR, PIK3CA, KIT, and platelet-derived growth factor receptor α polypeptide [PDGFRA]) by using next-generation sequencing (NGS) in a single thyroid tumor presenting as a combination of conventional PTC with 4 variants (follicular, clear cell, columnar and poorly differentiated [112,113,114,115]. While conventional PTCs showed only a limited rate of H/N/KRAS mutation (6%), the clear cell and the follicular variants harbored KRAS mutations up to 5 times more frequently, viz. 30% and 20% respectively [114,115,116]. Finally, in PTCs the degree of DNA methylation is smaller than in follicular tumors (FA and FTC), and it varies according to BRAF and RAS status [117].

Despite a robust line of research, clinical implications of ITH in PTCs are questioned. Indeed, two studies demonstrated that allelic frequencies of mutated alleles are consistent with a monoclonal origin of PTCs, suggesting ITH in as many as ~10% of tumors [117,118].

## 5. Conclusions

**Figure 6:**
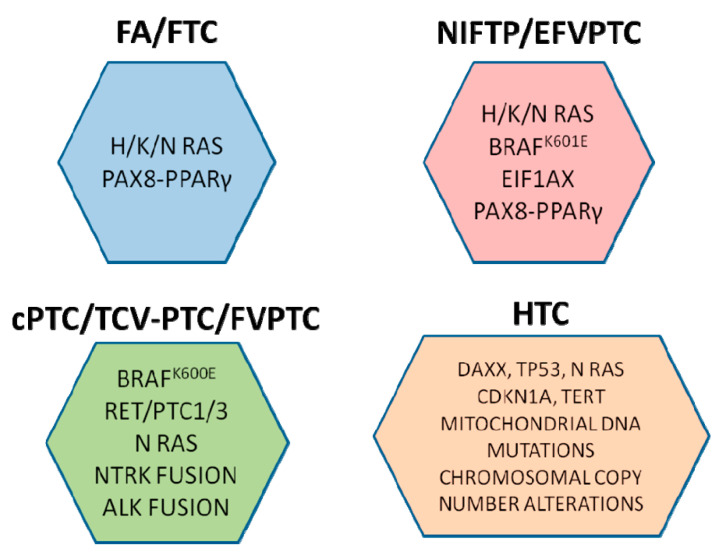
Synopsis showing the main biomolecular mutations in DTCs related to different histotypes.

ITH influences tumor progression and response to treatment, as the appearance of resistant clones due to the selection pressure of treatment may worsen the patient’s prognosis. Therefore, ITH profiling can be useful to characterize thyroid cancer pathogenesis, together with the analysis of different genetic alterations associated with oncological risk. Nowadays, molecular testing in DTCs suggests a high risk for recurrence of cancer associated with BRAF^V600E^, RET/PTC 1/3, ALK and NTRK fusions, while the intermediate risk may be related to BRAF^K601E^, H/K/N RAS and PAX8/PPARγ (Figure 6).

Consequently, it may be suggested that tumor genotype is associated with peculiar phenotype; therefore, the identification of DTC morphology may be the driver to select different neoplastic portions in which molecular heterogeneity could be revealed. From this point of view, neoplastic sub-populations with different risk of recurrence or metastasis may be advantageously stratified, correctly treated and subjected to a shorter follow-up period. Recently, to measure ITH in cancer, an index has been proposed [119]; in detail, some neoplasms such as uterine carcinosarcoma, colorectal adenocarcinoma and ovarian cancer have been observed to be more heterogeneous than renal clear cell carcinoma and DTC [119].

Recent studies based on genetic analysis of thyroid tumors have brought several intriguing therapeutical personalized options for DTCs [120]. This innovative vision in which particularly targeted therapies based on specific diagnostic tests has been defined as “theranostics”, in order to provide a transition from conventional to a contemporary personalized medicine [121]. The first theranostic agent has been considered as the radioiodine treatment widely used for the management of DTC. Nevertheless, about 65% of the patients with advanced thyroid disease may became radioiodine-refractory related to the sodium/iodide symporter (NIS) [120,121]. Therefore, the targeted therapy of DTC should be connected to the genetic and epigenetic alterations and signaling pathways. In detail, PPARγ agonists, HDAC inhibitors, PI3K/AKT inhibitors and MEK/ERK inhibitors, have been recommended for NIS over-expression and have caused improved iodine uptake in thyroid cancers [121]. Moreover, it was shown that Dabrafenib represents the selective inhibitor of mutated forms of *BRAF* and it can realize the radioiodine uptake in metastatic PTC *BRAF^V600E^*-mutant iodine-refractory patients. Similarly, some molecular markers such as *p53*, *PIK3CA*, *CTNNB1* and *AKT1* may be considered indicators for an aggressive behavior of DTCs [121]. Furthermore, since fine needle aspiration cytology (FNAC) has been considered the commonly utilized morphological test, the molecular profiling may improve the diagnostic accuracy mainly in indeterminate or gray zone, furtherly supporting a personalized treatment for DTCs [122].

A better understanding of the molecular basis of thyroid cancers as well as development of more effective cancer therapies has revolutionized the treatment approach in patients with advanced thyroid cancer. Nevertheless, whether overall survival is improved with the use of these agents is still unclear. In fact, the major limitation in applying targeted therapies is their side-effects profile, as well as in the development of escape and resistance mechanisms by the tumors. Specifically, neoplastic cells may acquire resistance to the treatment by developing an escape mechanism against the targeting drugs. Consequently, most DTCs could develop resistance against targeted drugs by acquiring new mutations that result in over-activation of pathways or by induction of alternate pathways.

Nowadays, the cancer diagnosis should be assessed by a complex of information regarding to clinical, pathological, molecular and protein expression data of a specific neoplastic proliferation and its surrounding microenvironment; such an integrated system has been defined as “*tissunomics*” [123,124]. In full agreement with this approach, we contend that a systematic integration of morphology and molecular characteristics in DTC should be helpful in patient’s management.

## Figures and Tables

**Table 1:** Histological and molecular heterogeneity in FA and follicular-patterned borderline lesions (H,K,N isoforms of RAS gene family).

	Mutations						
	PAX8-PPARγ	EIF1AX	EZH1	GNAS	RAS	BRAF	RET/PTC
FA	5–20%	5–10%	3%	~80%	HRAS 8%, KRAS 10%, NRAS 6%	-	-
NIFTP	5%	-	-	-	H/N/KRAS (45%)	-	-
HTT	-	-	-	-	NRAS 0%	-	47%
FT/WDT-UMP	<5%	-	-	-	HRAS 3–12%, KRAS 6–9%, NRAS 16–35%	-	-

**Table 2:** Histological and molecular heterogeneity in FTC and HCT.

	Mutations								
	PIK3CA	EIF1AX	TP53	PAX8-PPARγ	RAS	BRAF	TERTp	NF1	MADCAM-1
FTC	10%	6%	3%	10–50%	HRAS 8%, KRAS 6%, NRAS 19%	1%	17–25%	-	-
Poorly differentiated FTC	0–15%	-	10–30%	<3%	H/N/KRAS (10–40%)	5–30%	-	-	-
HCT	-	11%	7%	<5%	KRAS 11%NRAS 6%	-	17%	7%	20%

**Table 3:** Histological and molecular heterogeneity in PTC.

	Mutations							
	EIF1AX	TP53	RAS	BRAF	TERT	RET	ALK	NOTCH1
Classical PTC	0–5%	-	H/N/KRAS 6%	55%	5–15%	5–25%	-	-
Clear cell/solid variant PTC	-	-	H/N/KRAS 30%	>55%	-	-	5%	-
Columnar variant PTC	-	-	-	33%	-	-	-	-
Tall cell variant PTC	-	-	-	80–100%	-	-	-	-
Hobnail variant PTC	-	55%	-	25%	-	-	-	5%

## Data Availability

Not applicable.

## Abbreviations

AKT1	v-akt murine thymoma viral oncogene homolog 1
DTC	differentiated thyroid carcinoma
DTT	differentiated thyroid tumor
EGFR	epidermal growth factor receptor
EIF1AX	Factor 1A X-Linked
EZH1	zeste 1 polycomb repressive complex 2 subunit
FA	follicular adenoma
FTC	follicular thyroid carcinoma
GNAS	guanine nucleotide binding protein, alpha stimulating
HTT	hyalinizing trabecular tumor
ITH	intratumoral heterogeneity
MADCAM-1	Mucosal Vascular Address in Cell Adhesion Molecule 1
NF1	Neurofibromatosis type 1
NGS	next-generation sequencing
NIFTP	noninvasive follicular thyroid neoplasm with papillary-like nuclear features
NOTCH1	Notch homolog-1
PAX-8	paired box gene 8
PDGFRA	platelet-derived growth factor receptor α polypeptide
PI3K	Phosphoinositide 3-kinases
PPARγ	peroxisome proliferator-activated receptor-γ
PTC	papillary thyroid carcinoma
PTEN	Phosphatase and tensin homolog
RET	REarranged during Transfection
H,K,NRA	isoforms of RAS gene family
TERT	telomerase reverse transcriptase
TSHR	TSH-receptor
UMP	uncertain malignant potential tumor
WDT-UMP	well-differentiated tumors with uncertain malignant potential
